# Robert Langer and Mark Tibbitt answer questions about additive manufacturing

**DOI:** 10.1038/s41467-020-17724-1

**Published:** 2020-08-17

## Abstract

Robert S. Langer is an Institute Professor at the Massachusetts Institute of Technology. Leading one of the largest biomedical engineering labs in the world his research covers many areas of biotechnology including tissue engineering, drug delivery, biofabrication and the development of medical devices. Mark Tibbitt is an Assistant Professor of Macromolecular Engineering at ETH Zürich. His research focuses on combining polymer engineering, synthetic chemistry, mechanical and bioengineering for biofabrication, drug delivery and mechanobiology applications.

Tell us a little bit about you and what sparked your interest in additive manufacturing.

**Bob:** I like to think that our lab at MIT has made pioneering contributions in two cores areas of biomedical engineering: tissue engineering and drug delivery. We are always seeking out emerging technologies, like additive manufacturing, that can advance these paradigms and accelerate our impact on human health. We became interested in additive manufacturing in the context of engineering personalized implants, standardizing microtissues for disease modeling and pharmaceutical development, and fabricating controlled release technologies. This work builds upon paradigms we established in the field and exploits new processing, design, and fabrication opportunities afforded by additive manufacturing.

**Mark:** I joined the Langer Lab as a postdoc in 2013 and at this time the benefit of additive manufacturing for biomedical engineering was becoming more apparent, especially for the design of personalized or precision biomaterials. We first applied additive manufacturing in the lab in a project with Héloise Ragelle, another former postdoc of the Langer Lab, to design and manufacture a multi-material tracheal stent to help cardiothoracic surgeons looking to repair or replace damage tracheal tissue^1^. We designed lattice tubes using stereolithography to meet the geometric and mechanical needs of the stent, which would not have been feasible using another manufacturing technique, and developed a process to use surface tension to form a cell-laden hydrogel film around the stent, to introduce biofunctionality to the device. In my lab at ETH Zürich, we are currently designing materials (or inks) for additive manufacturing that can be used to fabricate personalized tissue models and drug delivery systems.

How has additive manufacturing changed your field? Is there anything specific that it has enabled or made possible? Tell us how it impacted your research.

Additive manufacturing is a transformative enabling technology that provides researchers across many domains improved access to design the form and function of the materials they produce. We are already seeing the impact of additive manufacturing in the design and development of commercial products as it transitions from a technology for prototyping to a means of production. In biomedical engineering, additive manufacturing offers specific advantages for both tissue engineering and drug delivery as it enables access to complex structures and detailed geometries that are often not possible (or cost-prohibitive) with traditional subtractive manufacturing. Further, additive manufacturing uses digital design for high precision fabrication, which can enable the production of personalized implants based on medical imaging.

One recent example of the impact of additive manufacturing is the recent demonstration of complex vascularized tissue constructs enabled by advanced stereolithography^2^. Vascularization of human-scale constructs remains a major challenge in the field of tissue engineering and additive manufacturing may be one solution that addresses this challenge.

In our lab, additive manufacturing has been used, as Mark mentioned, to design multi-material structures to help surgeons design better tracheal stents. We have also leveraged bioprinting to generate reproducible 3D tissue models to investigate disease processes and for drug testing ex vivo. This includes the design of microscale cardiac valve models, in collaboration with Elena Aikawa at Harvard Medical School, to study calcific valve disease^3^. We have also applied the technology to drug delivery and medical device design, Yong Kong and Giovanni Traverso used additive processes in the Langer Lab to design gastric resident devices^4^. Kevin McHugh, Thanh Nguyen, and Ana Jaklenec developed a new additive process, StampEd Assembly of polymer Layers or SEAL, to engineer single-injection vaccinations^5^.

What does additive manufacturing have to do to be more widely adopted? Are there any specific hurdles it has to overcome?

While additive manufacturing has already advanced our ability to design complex yet precise biomaterials, there are several open problems that should be addressed to increase its adoption and clinical use. A core area of focus remains on the technology itself. Often additive processes are limited in the scale and speed of production as well as the resolution and complexity of the final part. Technologies like CLIP, commercialized by carbon, volumetric additive manufacturing, and multi-material printing have improved the speed, resolution, and complexity of additively manufactured products^6–8^. Yet, further developments are needed to realize intricate, multi-cellular tissues of arbitrary complexity, rapid production of patient-specific implants, and the design of personalized pharmaceutical products.

Another current challenge is the range of biomaterials suitable for additive processes. The biomaterials community has made tremendous advances and many GRAS-listed and FDA-approved materials exist for the design of medical devices. Many of these are suitable for additive processes, but not all. We will need to continue working closely with engineers, materials scientists, and clinicians to develop the next-generation of compatible biomaterials that integrate with additive processes.

A further challenge in the broad adoption of additive manufacturing in the biomedical sciences is the pathway to regulatory approval. Regulatory agencies, including the FDA, have provided guidelines for how additive manufacturing will be considered in the regulatory process and already some products have been approved. However, uncertainties remain about how clinical production would operate for additively manufactured biomedical products and whether this would require centralized production facilities, as in standard medical device fabrication, or if technologies will be approved as decentralized production facilities in individual care centers or pharmacies.

Looking forward: where do you see additive manufacturing going next?

We see that additive manufacturing will have the most impact in the field of biomedical engineering where the benefits of digital design, rapid production, and personalized are needed. In the future, this may include the design of complex tissue constructs with spatial resolution at the single cell level containing both vascularization and innervation. Digital design and fabrication based on medical imaging will likely advance the paradigm of precision biomaterials that are built specifically to address individual patients or subsets of patients. Finally, an emerging use of additive manufacturing is on the design of personalized drug delivery systems^9^, and future pharmaceutical development may combine controlled release technologies with additive manufacturing to further enable personalized drug delivery.

For you, is there a difference between additive manufacturing and 3D printing? If they are not equivalent, how do they differ?

The definitions of these terms are not very precise, but one view is that 3D printing describes processes that build 3D structures through successive layer-by-layer formation, like stacking many prints into a 3D object. Whereas additive manufacturing is a more comprehensive term that describes all processes that can be used to fabricate 3D forms by adding, combining, or joining material components, including those that are beyond layer-by-layer, in contrast to traditional subtractive manufacturing where material is removed to produce the final part. Both terms describe methods to produce 3D objects and in practice they have often become synonymous.
